# Cytogenetic Analysis of Amniotic Fluid Cells in 4206 Cases of High-Risk Pregnant Women

**Published:** 2019-01

**Authors:** Huafeng LI, Yongli LI, Rui ZHAO, Yanli ZHANG

**Affiliations:** Genetic Laboratory, Women & Children’s Health Care Hospital of Linyi, Shandong Province, Linyi, 276014, PR China

**Keywords:** Chromosomal karyotype, Chromosomal abnormalities, Amniocentisis, Prenatal diagnosis

## Abstract

**Background:**

We aimed to assess the frequency and structure of chromosomal abnormalities as well as the distribution of the indications of prenatal diagnosis in 4206 cases of high-risk pregnant women.

**Methods:**

A retrospective analysis of cytogenetic studies of 4206 pregnant women with indications of amniocentesis, referred to Linyi Women and Children’s Hospital, Shandong Province, Linyi, China in 2016–2017, was performed. Among those, 4191 amniotic fluid specimens were successfully extracted and cultured, and received karyotype diagnosis.

**Results:**

A total of 358 abnormal karyotypes were detected and the abnormal rate was 8.54%. Among them, autosomal aneuploidy was the most common pattern occupied 64.53% and the detection rate was 5.51%, of which 173 (48.32%) cases were 21-trisomy, which was the main type of abnormal karyotypes, followed by 18-trisomy (14.25%). There were 38 cases with sex chromosome aneuploidy, including 47, XXY, 47, XXX, 47, XYY, 69, XXX and 45, X0, accounting for 10.61% of the total chromosome abnormalities and the detection rate was 0.91%. Chromosome structural disorders occupied 10.61% (38/358) of the chromosome abnormalities, including Robertson translocation (16 cases), balance translocation (14 cases), inversion (3 cases), deletion (3 cases), and so on. Chromosome polymorphism was 10.61% too. Other uncommon abnormal karyotypes included mosaicism (11/358), marker chromosome (1.3%). Advanced age and serological screening for high risk were the major prenatal diagnostic indications for pregnant women with chromosomal abnormalities.

**Conclusion:**

The karyotype analysis of amniotic fluid cells in pregnant women with different amniocentisis indications can effectively prevent the birth of fetuses with chromosomal diseases and reduce the risk of fetal malformation.

## Introduction

Chromosomal abnormalities are one of the common causes of neonatal birth defects, characterized by intellectual disability, multiple malformations and so on. There are no effective treatments for fetal abnormalities at present, which brings heavy economic pressure and psychological burden on families and society (1).

Prenatal screening and diagnoses are effective means to direct aristogenesis and good brood, which can availably reduce the born of children with chromosomal diseases. The karyotype analysis of amniotic fluid cells was the main method for detecting fetal chromosomal abnormalities and was regard as the gold standard for cytogenetic diagnosis currently.

We examined 4206 cases of high-risk pregnancies by amniocentesis diagnostic technique. We were able to summarize the correlation between chromosomal abnormalities and various indications of prenatal diagnosis during pregnancy.

## Materials and Methods

### Subjects

A total of 4206 pregnant women with indications of prenatal diagnosis came to Genetic laboratory and prenatal diagnosis center of Women & Children’s Health Care Hospital of Linyi, China form January 2016 to December 2017. They were performed amniocentesis under informed consent. The indications of prenatal diagnostic include: advanced maternal age, high-risk serological screening, abnormal non-invasive prenatal DNA test, ultrasonographic abnormal indications, paternal/maternal carrying chromosome abnormality, a history of intrauterine fetal death or aborted fetuses. The maternal age was range from 15 to 49 yr and the gestational week was range from 16 to 31weeks.

### Methods

Detailed genetic counseling and informed consent was performed on pregnant women before amniocentesis. Amniocentesis was performed aseptically under the guidance of ultrasonography. Twenty milliliter of amniotic fluid was collected and centrifuged at 1500 r/min for 10 minutes. The supernatant was discarded after centrifugation, leaving about 1–2 ml of cell suspension.

Added 5 ml of amniocyte culture medium (Gibco, USA and Israel) and grew in an incubator at 37 °C and 5% CO2 for 9 to 10 days. Cell growth was monitored daily. The amniotic fluid cells were harvested when multiple clones with numerous metaphase cells were observed under an inverted microscope. Conventional G banding was performed and then scanned by Leica GLS120 Automated Nuclear Scanning System. Twenty chromosome karyotypes were counted and 5 karyo-types were analyzed by two doctors according to the ISCN 2009 standard. Lymphocyte karyotype analysis of the couples is recommended if the chromosome structure is abnormal.

## Results

### The classification and detection rate of abnormal karyotypes

Overall, 4191 amniotic fluid specimens were successfully extracted and cultured among 4206 cases and the success rate was 99.64%. Fifteen cases had failed to culture because of low amniotic fluid volume and turbid amniotic fluid. A total of 358 chromosomal abnormalities were detected in 4191 fetuses and the detection rate was 8.54% (358/4191). Of the 358 fetuses, aneuploidy was the most common pattern which was up to 75.14% (269/358) and the detected rate was 6.42% (269/4191), the most common type was 21-trisomy (173/358, 48.32%), followed by 18-trisomy (51/358, 14.25%). Sex aneuploidy made up 10.61% (38/358) of chromosomal abnormalities and included 47, XXY (17/358), 47, XXX (11/358), 47, XYY(4/358), 69,XXX (3/358), 45,X (3/358) (Table 1). The structural disorders of chromosome were detected in 10.61% (38/358) patients: balanced structural chromosomal rearrangements (33 cases) prevailed over non-balanced structural rearrangements (5 cases) significantly (Table 2). Others included chromo-some polymorphisms (38/358, 10.61%), mosaicism (11/358, 3.07%) and marker chromosomes (2/358, 0.56%) (Table 3).

**Table 1:** The classification and detection rate of 358 chromosomal abnormal karyotypes

***Chromosomal karyotype***	***Number (n)***	***% Occupancy (n/358)***	***% Detection rate (n/4191)***
47,XX/XY,+21	173	48.32	4.13
47,XX/XY,+18	51	14.25	1.22
47,XX/XY,+13	7	1.96	0.17
47,XXX	11	3.07	0.26
47,XYY	4	1.12	0.10
47,XXY	17	4.75	0.41
69,XXX	3	0.84	0.07
45,X	3	0.84	0.07
Structural abnormality	38	10.61	0.91
Mosaic	11	3.07	0.26
Chromosome polymorphism	38	10.61	0.91
Marker chromosome	2	0.56	0.05
Sum	358	100	8.54

**Table 2:** The karyotypes of 38 structural disorders of chromosome

***Type Robertson translocation***	***Karyotype***	***Numbers 16***	***Type Balance translocation***	***Karyotype***	***Number*** ***14***
	45,XY,rob(13;14)(q10;q10)	6		46,XY,t(2;10)(q31;q24)	1
	45,XX,rob(14;21)(q10;q10)	2		45,XY,t(14;20)	1
	46,XY,rob(21;21)(q10;q10)	3		46,XY,t(3;11)(q27;q13)	1
	45,XX,rob(13;15)(q10;q10)	1		46,XX,t(2:3)(q23;p23)	1
	46,XX,rob(14;21)(q10;q10),+21	2		46,XY,t(2;7)(q13;q36)	1
	46,XY,rob(21;21)(q10;q10),+21	1		46,XY,t(6;18)(q21;q23)	1
	45,XX,rob(13;22)(q10;q10)	1		46,XY,t(7;19)(q11.1;q12)	1
Inversion		3		46,XY,t(2;3)(p11.2;p14)	1
	46,XY,inv(1)(p13.3q25)	1		46,XX,t(1;18)(q25;p11.2)	1
	46,XX,inv(2)(p11.2q13)	1		46,XX,t(13;22)(q21;p12)	1
	46,XX,inv(3)(p23q21)	1		46,XY,t(4;8)(q33;q11.2)	1
Deletion		3		46,XX,t(14;15)(q24;q11.2)	1
	46,X,del(X)(q21)	1		46,XX,t(3;10)(q26.2;q22)	1
	46,XY,del(18)(p11.2)	1		46,XY,t(4;14)(q31.1;q24)	1
	46,X,del(X)(p11.2)	1	Others		1
Deletion		1		46,XY,12p?	1
Duplication	46,XY,dup(1)(q42.1q44)	1			

**Table 3:** Other karyotypes of chromosomal abnormalities

***Type***	***Karyotype***	***Number***	***Type***	***Karyotype***	***Number***
Chromosome polymorphism		38	Mosaic		11
	46,XX,inv(9)(p11q13)	15		46,XX[41]/45,X[25]	1
	46,XX/XY, 1qh+	5		46,XY[53]/47,XY+21[8]	1
	46,XX/XY, 13pstk+	1		47,XXY[54]/46,XY[6]	1
	46,XX/XY, 13centh+	1		46,XY,inv(8)[13]/46,XY[22]	1
	46,XX/XY, 13ps+	1		47,XX,+13[64]/48,XX,+12,13[34]	1
	46,XX/XY, 14 centh+	1		46,XY[73]/47,XY+21[17]	1
	46,XX/XY, 14ps+	1		46,XX[70]/46,XX,rob(21;21),+21[5]	1
	46,XX/XY, 15 centh+	2		47,XX,+13[71]/46,XX[10]	1
	46,XX/XY, 15 pstk+	1		47,XXY[32]/46,XY[41]	1
	46,XX/XY, 15ps+	2		47,XX,+18[32]/46,XX[30]	1
	46,XX/XY, 16qh+	2		47,XXX[7]/46,XX[53]	1
	46,XX/XY, 21 centh+	2	marker chromosomes	47,XX/XY,+mar,	2
	46,XX/XY, 21ps+	1			
	46,XX/XY, 22 centh+	1			
	46,XX/XY, 22ps+	2			

### The distribution of the indications of prenatal diagnosis in chromosomal abnormalities

Advanced age and serological screening were the main indications of prenatal diagnosis in 358 chromosomal abnormalities. Autosomal aneuploidy was the most common in advanced maternal age. The pregnant women with advanced age reached 71.68% (124/173) and 70.58% (36/51) in 173 cases with 21-trisomy and 51cases with 18-trisomy respectively.

Sex chromosome abnormalities were concentrated in non-invasive prenatal DNA group (18/38, 47.37%), followed by advanced age (9/38, 23.68%).

The structural of chromosomal abnormalities were concentrated in high-risk serological screening (14/38, 36.84%) and paternal/maternal abnormality group (10/38, 26.32%). Mosaicism and chromosome polymorphism mainly distributed in the high-risk serological screening group, accounting for 63.64% (7/11) and 68.42% (26/38) of abnormalities in this group (Table 4).

**Table 4:** The distribution of high-risk indications in 358 chromosomal abnormalities

			The indications of prenatal diagnosis				
***Chromosomal karyotype***	***High-risk serological screening***	***Advanced maternal age***	***Abnormal ultrasonographic indications***	***Abnormal non-invasive prenatal DNA test***	***Paternal/maternal carrying chromosome abnormality***	***A history of intrauterine fetal death or aborted fetuses***	***Sum***
47,XX/XY,+21	22	124	9	18			173
47,XX/XY,+18	13	36	1	1			51
47,XX/XY,+13	2	1	1	3			7
47,XXX		4		7			11
47,XXY	3	3		11			17
47,XYY	2	1	1				4
69,XXX	3						3
45,X	1	1	1				3
Structural abnormality	14	3	2	5	10	4	38
Mosaic	7			4			11
Chromosome polymorphism	26	7	1	1	2	1	38
Marker chromosome		1		1			2
Sum	93	181	16	51	12	5	358

Note: If the pregnant women fulfilled advanced age with other indications of prenatal diagnosis simultaneously, classified as advanced maternal age group

## Discussion

Chromosomal abnormalities are the common genetic disorders caused neonatal birth defects. The incidence of chromosomal abnormalities is about 0.5% in live newborns (2), which reached 5% to 13% in stillbirths (3). There are no effective treatments for fetal abnormalities currently. The karyotype analysis of amniotic fluid cells in second trimester is an important preventive mean for prenatal diagnosis and timely termination of aberrant pregnancies (4).

Our study performed 4191cases of high-risk pregnancies by amniocentesis diagnostic technique successfully. A total of 358 abnormal karyotypes were found among 4191 fetuses, and the abnormal rate was 8.54% (358/4206). Autosomal aneuploidy was the most common pattern occupied 64.53% (231/358) and the detection rate was 5.51% (231/4191), the most common karyo-type was 21-trisomy (173/358, 48.32%). Termination of pregnancy is recommended because of trisomy syndrome with definite pathogenicity. There are 38 cases of sex aneuploidy, made up 10.61% (38/358) of chromosomal abnormalities. The fetuses of 47, XXY caused by abnormal separation of the paternal sperm cells and the maternal ovum cells, which characterized by testicular developmental disorder and infertility. The majority of 47, XYY patients had normal fertility and secondary sexual characteristics. 47, XXX have the normal fertility and phenotype. 45, X, also known as congenital ovarian hypoplasia syndrome, is a common cause of female primary closure. For fetuses with abnormal sex chromosomes, the couple should be informed of the possible risk of infertility in adulthood, allowing them to make informed choices.

The structural disorders of chromosome were observed in 38 of 358 fetuses: including balanced structural chromosomal rearrangements (33 cases): balanced translocation (14 cases), Robert-sonian translocation (16 cases), inversion (3 cases); and non-balanced structural rearrangements (5 cases) including deletion (3 cases), repeat (1 cases), and 1 case abnormal short arm of chromosome 12. In order to help judging the origin of abnormal karyotype, some parents were required to analyze lymphocyte karyotype.

Ten familial and 1 de novo abnormalities were found in parents of 14 cases of balanced translocation fetuses, the rest of 3cases followed failure. Three cases complicated with 21-trisomy syndrome were advised termination of pregnancy in 16 Robertsonian translocation fetuses, others were familial inheritance. One case of inversion was inherited from the fetus’ mother, and 1 was de novo inversion, the rest was followed failure. According to the traditional cytogenetics, it was considered that balanced structural chromosomal rearrangements without involving the missing of genes do not cause clinical symptoms generally, so it was advised to continue the pregnancy (5). However, the couples should be informed the possible risk of infertility in adulthood.

Four cases in 5 unbalanced structural rearrangements accepted the further test of chromosome microarray analysis (CMA). The results showed that 46,X,del (18) (p11.2) had a 14.9 Mb fragment loss in p11.32p11.21 region. 46,X,del (X) (q21) was detected a 67Mb fragment loss in q21.31q28 region of the X chromosome, and a duplication of 24.3 Mb fragment was present in q42.11q44 region of chromosome 1, all of which suggested intellectual disability and global development delay of fetuses. 46, X, del (X) (q21) also have clinical manifestations of Turner syndrome. The CMA of the terminal of short arm of chromo-some 12 showed a13.9Mb duplication in q22.1q23 region of chromosome 18 and a 1.2 Mb deletion in p13.33 region of chromosome 12, which suggested an unbalanced translocation of chromosome 12 and 18. Duplicated segment of chromosome 18 may cause intellectual disability, global development delay, and abnormal facial shape. Segment 12 deletions are associated with neurological disorders. It’s generally acknowledged that there were deletions or duplications of the genes in unbalanced structural rearrangements. It was advised to terminate the pregnancy and perform the prenatal diagnosis at the next pregnancy.

In our study, 38 cases of chromosome polymorphisms were found in 358, including 15 cases of pericentric inversion of chromosome 9, 16 cases of increasing in length of the heterochromatin on the centromere and satellite of D/G group (13/14/15/21/22) chromosome, 7 cases of increasing in length of the heterochromatin on the long arm of a chromosome1/16, which were considered normal variations and believed that there were no obvious clinical phenotypic effects generally (6). The pericentric inversion of chromosome 9 is a relatively common chromosome structure variation and occurs in 0.82% of the population (5). The incidence of chromosome 9 inversion was detected 0.36% in our study, which was similar with detection rate of 0.55% (7).

Chromosome mosaicism refers to the presence of two or more different karyotypes in the same body. Mosaicism can be divided into true mosaicism and pseudo- mosaicism. Pseudo- mosaicism and maternal blood contamination can be excluded by chromosome examination of umbilical cord blood. In this study, 11 cases of mosaicism were detected, of which 7 cases had induced labor and 2 cases agreed to perform umbilical cord blood tests and the results showed normal karyo-type and 2 cases followed up loss.

Advanced age and high risk of serological screening were the main indications for genetic amniocentesis in 358 chromosomal abnormalities. The risk of fetal chromosomal diseases increased because of the maternal ovum aging and chromo-some non-segregation of pregnant women with advanced age (8). Advanced age accounted for 50.56% (181/358) in 358 cases of chromosomal abnormalities, of which 124 cases were 21-trisomy and 36 cases were 18-trisomy, accounting for 71.68% (124/173) of 21-trisomy and 70.59% (36/51) of 18-trisomy. It should be concerned that 133 pregnant women were detected of high risk of non-invasive prenatal DNA test or suggesting other chromosome abnormalities in 181 pregnant women with advanced age, which of 117 cases of high risk of aneuploidy, 9 cases of sex chromosome abnormalities and 3 cases of other chromosomal abnormalities in 133. It can be seen that non-invasive DNA prenatal detection not only has high accuracy in 21-trisomy and 18-trisomy but also provide a suggesting on sex chromosomes and other chromosome abnormalities and accepted by majority of pregnant women because of its advantages of high accuracy, non-invasiveness and low-risk. Fetal abnormal karyotype was found in 93 pregnant women with abnormal maternal serum screening tests. Of the 93 pregnant women, 37 cases had 13, 18 or 21-trisomy, 14 cases observed structural abnormalities, 26 cases showed chromosome polymorphism (9). Positive serum screening results could not only suggest trisomy syndrome, but also other chromosome diseases, which was confirmed by Xu Ling-ling et al who reported 54 pregnant women with high risk of serological screening in 101 chromosomal structural disorders (10).

## Conclusion

Karyotype analysis of amniotic fluid is an important approach to prevent the birth of fetuses with chromosomal disease. Our results highlight the importance of cytogenetic studies in patients with indications of prenatal diagnosis, since an abnormal finding not only provides the option of termination or continuation of the pregnancy, but also constitutes a basis for genetic counseling.

## Ethical considerations

Ethical issues (Including plagiarism, informed consent, misconduct, data fabrication and/or falsification, double publication and/or submission, redundancy, etc.) have been completely observed by the authors.

## References

[1] Templado C Bosch M Benet J (2005). Frequency and distribution of chromosome abnormalities in human spermatozoa. Cytogenet Genome Res, 111: 199– 205. 16192695 10.1159/000086890

[2] Hook EB Schreinemachers DM Willey AM Cross PK (1984). Inherited structural cytogenetic abnormalities detected incidentally in fetuses diagnosed prenatally: frequency, parental-age associations, sex-ratio trends, and comparisons with rates of mutants. Am J Hum Genet, 36: 422– 443 6711562 PMC1684413

[3] Reddy UM Goldenberg R Silver R (2009). Stillbirth classification--developing an international consensus for research: executive summary of a National Institute of Child Health and Human Development workshop. Obstet Gynecol, 114: 901– 914. 19888051 10.1097/AOG.0b013e3181b8f6e4 PMC2792738

[4] Vaknin Z Reish O Ben-Ami I Heyman E Herman A Maymon R (2008). Prenatal diagnosis of sex chromosome abnormalities: the 8-year experience of a single medical center. Fetal Diagn Ther, 23: 76– 81. 17934303 10.1159/000109231

[5] Hulten MA Dhanjal S Pertl B (2003). Rapid and simple prenatal diagnosis of common chromosome disorders: advantages and disadvantages of the molecular methods FISH and QF-PCR. Reproduction, 126: 279– 297. 12968936 10.1530/rep.0.1260279

[6] Schluth-Bolard C Delobel B Sanlaville D (2009). Cryptic genomic imbalances in de novo and inherited apparently balanced chromosomal rearrangements: array CGH study of 47 unrelated cases. Eur J Med Genet, 52: 291– 296. 19505601 10.1016/j.ejmg.2009.05.011

[7] Zhang YP Wu JP Li XT Lei CX Xu JZ Yin M (2011). [Karyotype analysis of amniotic fluid cells and comparison of chromosomal abnormality rate during second trimester]. Zhonghua Fu Chan Ke Za Zhi, 46: 644– 648 [Article in Chinese]. 22176986

[8] Ogilvie CM Lashwood A Chitty L Waters JJ Scriven PN Flinter F (2005). The future of prenatal diagnosis: rapid testing or full karyo-type? An audit of chromosome abnormalities and pregnancy outcomes for women referred for Down’s syndrome testing. BJOG, 112: 1369– 1375. 16167939 10.1111/j.1471-0528.2005.00695.x

[9] Zhang L Zhang XH Liang MY Ren MH (2010). Prenatal cytogenetic diagnosis study of 2782 cases of high-risk pregnant women. Chin Med J (Engl), 123: 423– 430. 20193481

[10] Zhong SL Fang Q Chen BJ Han ZY Luo YM Chen JS Xie YJ (2011). [Clinical features of abnormal chromosome karyotypes in twin pregnancies complicated with structural abnormalities]. Zhonghua Fu Chan Ke Za Zhi, 46: 649– 654 [Article in Chinese]. 22176987

